# Let’s Tie the Knot: Marriage of Complement and Adaptive Immunity in Pathogen Evasion, for Better or Worse

**DOI:** 10.3389/fmicb.2017.00089

**Published:** 2017-01-31

**Authors:** Kaila M. Bennett, Suzan H. M. Rooijakkers, Ronald D. Gorham

**Affiliations:** Department of Medical Microbiology, University Medical Center UtrechtUtrecht, Netherlands

**Keywords:** complement system, adaptive immunity, immune evasion, crosstalk, antigen presenting cell, complement receptors

## Abstract

The complement system is typically regarded as an effector arm of innate immunity, leading to recognition and killing of microbial invaders in body fluids. Consequently, pathogens have engaged in an arms race, evolving molecules that can interfere with proper complement responses. However, complement is no longer viewed as an isolated system, and links with other immune mechanisms are continually being discovered. Complement forms an important bridge between innate and adaptive immunity. While its roles in innate immunity are well-documented, its function in adaptive immunity is less characterized. Therefore, it is no surprise that the field of pathogenic complement evasion has focused on blockade of innate effector functions, while potential inhibition of adaptive immune responses (via complement) has been overlooked to a certain extent. In this review, we highlight past and recent developments on the involvement of complement in the adaptive immune response. We discuss the mechanisms by which complement aids in lymphocyte stimulation and regulation, as well as in antigen presentation. In addition, we discuss microbial complement evasion strategies, and highlight specific examples in the context of adaptive immune responses. These emerging ties between complement and adaptive immunity provide a catalyst for future discovery in not only the field of adaptive immune evasion but in elucidating new roles of complement.

## Introduction

The complement system is an evolutionarily conserved branch of innate immunity, which is comprised of a proteolytic cascade of numerous proteins in serum. Complement is historically regarded as a first line of defense against invading pathogens, and activation of the enzymatic cascade leads to their rapid recognition and elimination. Activation of this cascade leads to leukocyte chemotaxis, phagocytosis, and direct cell lysis via the MAC. While complement has long been regarded as an isolated immunological pathway, mounting recent evidence ties complement to many other physiological processes.

Complement is considered an important link between innate and adaptive immunity. While its roles in innate immunity have been well-characterized, we are only beginning to understand the complex “crosstalk” between complement and the adaptive immune system. Historically, complement’s only established role in adaptive immunity was its function in humoral antibody response, in which complement opsonization reduces the B cell activation threshold in response to antigens, subsequently leading to increased antibody production. Since the initial discovery more than 40 years ago, many new roles have been described, including antigen capture by FDCs and antigen presentation. In the last decade, numerous studies have elucidated direct roles of complement in T cell immunity. Moreover, new mechanisms are continually being identified, suggesting an increasingly crucial role of complement in directing adaptive immune responses.

While the human immune system is highly evolved to mount an effective response to nearly any encountered threat, invading pathogens do not give up without a fight. In a struggle for survival, pathogens and their human hosts are engaged in a perpetual arms race. Indeed, many immune evasion strategies have been discovered in recent decades, and much work has focused on complement evasion by human pathogens (reviewed in Lambris et al., 2008; Stoermer and Morrison, 2011; Ricklin, 2012; Zipfel et al., 2013; Merle et al., 2015b; Garcia et al., 2016). Many of the goals of pathogenic complement evasion are clear, particularly inhibition of phagocytosis and direct killing by MAC. However, the role of complement in directing adaptive immune responses against pathogens is only beginning to be understood, and how pathogens circumvent these processes is less clear.

Herein, we describe how pathogens modulate adaptive immunity through complement regulation. We highlight the known mechanisms by which complement drives adaptive immune responses, and summarize evasion strategies used by pathogens to direct adaptive immunity through complement engagement. Furthermore, we postulate generalized mechanisms that pathogens can employ to subvert complement-mediated B and T cell responses.

## Complement Activation

**FIGURE 1:**
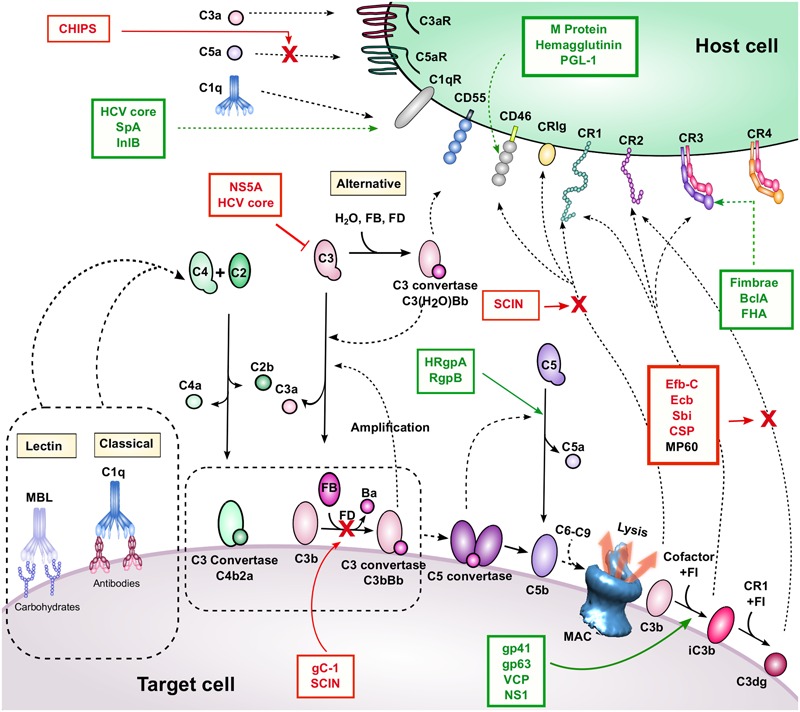
**Complement activation, regulation, and inhibition.** The three activation pathways are shown near the corresponding yellow-shaded boxes (classical, lectin, and APs). These pathways lead to C3b and C4b deposition, convertase, and MAC formation on the target cell surface. Breakdown mediated by complement regulators leads to iC3b and C3dg formation. Complement receptors and regulators are depicted on the host cell surface. Interactions are indicated by dashed black arrows, and enzymatic cleavage events are indicated by solid black arrows. Proteins listed in red boxes are immune evasion molecules that inhibit the complement interactions indicated by the solid red arrow and red “X”. It is unknown whether MP60 (in black font) inhibits the C3d-CR2 interaction, although it binds to C3d. A red inhibitory line indicates inhibition of protein expression. Proteins listed in green boxes with dashed green arrows bind to the indicated complement protein, and those with solid green arrows facilitate the indicated cleavage event.

Complement activation proceeds via three pathways (classical, lectin, and alternative) that, while activated via different mechanisms, all lead to cleavage of C3, the central component of the complement cascade, and the effector functions described above (**Figure 1**). These mechanisms are briefly described here, but are reviewed in detail elsewhere (Merle et al., 2015a). The CP is initiated by antigen-bound IgG or IgM, which allows binding of the C1 complex (C1qrs). The protease component, C1s, then cleaves C4 and C2, to form a C3 convertase (C4b2a) on a target surface. The LP is activated in a similar manner, but rather than relying on antibodies, MBL recognizes carbohydrate patterns conserved among microorganisms. This binding event activates MASPs, which, similarly to C1s, cleave C4 and C2 to form C4b2a. The AP is spontaneously activated by hydrolysis of C3 to form C3(H_2_O), which can form a complex with FB. Upon cleavage by FD, a C3 convertase is formed [C3(H_2_O)Bb], which like C4b2a, can mediate cleavage of C3 into C3a and C3b. The newly formed C3b molecule can also form an AP C3 convertase enzyme (C3bBb) similarly to C3(H_2_O). Upon cleavage by C3 convertase enzymes, C3 undergoes a large conformational change, whereby its intrinsic thioester becomes exposed and can quickly react with molecules on cell surfaces. This reaction mediates covalent cell-surface attachment of the activated cleavage product C3b. Since C3b comprises the C3 convertase of the AP, C3b deposition initiates an amplification loop, which leads to rapid opsonization of the target cell surface. Opsonization by C3b leads to several functional outcomes. High densities of C3b on cell surfaces can lead to formation of C5 convertase enzymes, which cleave C5 into C5a and C5b. This event initiates formation of MAC, which can directly mediate cell lysis. In addition, the ATs C3a and C5a are involved in chemoattraction of leukocytes during infection, and have been shown to play emerging roles in regulating T cell immunity (discussed in detail below).

Complement also has numerous regulatory mechanisms to control complement activation, particularly on host cells where complement activation is undesirable. Indeed, many host cells express CD55 (DAF), which dissociates C3 convertases and prevents further amplification. In addition, CD46 (MCP) and CR1 (CD35) bind to C3b and serve as cofactors for FI to cleave C3b to iC3b, which is no longer able to form active convertase enzymes. Thus, complement activation and amplification are slowed. These regulatory events are also mediated by FH, a soluble complement regulator that binds to anionic markers on host cell surfaces. Likewise, C4BP, a soluble regulator of the CP and LP, facilitates FI-mediated cleavage of C4b.

Breakdown of C3b plays an important role in pathogen clearance and downstream adaptive immune response. C3b is recognized by CR1, which is involved in clearance of target cells and immune complexes from the bloodstream. Concomitantly, CR1 facilitates cleavage of C3b to iC3b [and eventually to C3d(g)], which can also interact with several CRs on cell surfaces (including CR2, CR3, and CR4), participating in a variety of functions including phagocytosis, antigen presentation, and lymphocyte stimulation.

## Complement Regulation of Adaptive Immunity

### C3d-CR2 Interaction and B Cell Immunity

**FIGURE 2:**
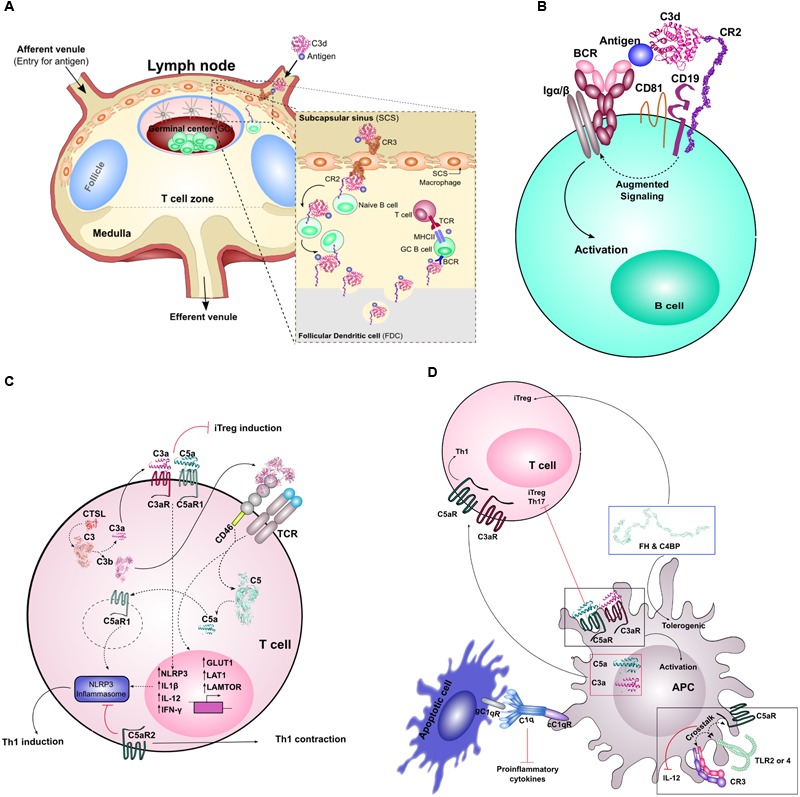
**Crosstalk between complement and adaptive immunity. (A)** Depicts the entry of C3d-coated immune complexes into the lymph nodes via the afferent venule. These complexes will associate with CR3 on subcapsular macrophages. These complexes will move from the apical side to basolateral where the immune complex is transferred to CR2 on the surface of naïve B cells within the GC, and handed off to FDCs. In the final stages, this complex is then presented to affinity matured GC B cells for T cell dependent responses. **(B)** Highlights the molecular adjuvant affect of C3d. In low antigen conditions, antigen engagement of BCR alone is not sufficient to promote associated Igα/β signaling and B cell activation. Antigen-bound C3d will bind CR2, part of the B cell co-receptor complex (together with CD19 and CD81), and coligation of BCR and the B cell co-receptor complex amplifies the molecular signal that leads to downstream B cell activation. **(C)** Illustrates the role of locally and intracellularly synthesized complement components on T cell homeostasis and priming. Intracellular C3 is cleaved by CTSL into biologically active C3a and C3b. Intracellular C3a/C3aR association is important for T cell survival. Extracellular C3a/C3aR and C5a/C5aR association influence Th1 lineage commitment, and in their absence, can lead to iTreg induction. The extracellular association of C3b with its receptor CD46 on T cells influences the metabolic state of the T cell, shifting it from a metabolically quiescent state to an active state (through increased GLUT1, LAT1, and LAMTOR expression). Costimulation of CD46 upon activation of TCR influences the expression of NLRP3 and results in cleavage of intracellular C5. Extracellular C5aR2 activation provides signals that inhibit the activation of the NLRP3 inflammasome leading to increased IL-10 production and Th1 contraction. Paradoxically, intracellular C5aR1, upon activation, leads to activation of the inflammasome resulting in Th1 induction. **(D)** Highlights the role of complement in activation of APCs and their crosstalk with T cells. Activation of extracellular C3a/C3aR and C5a/C5aR results in the activation of the APC. APC generated C3a and C5a can bind to the AT receptors on T cells to induce a Th1 response. Absence of C5aR on DCs leads to the induction of Th17 and Tregs. Extracellular stimulation of APCs, primarily DCs, with complement soluble regulators C4BP and FH leads to a tolerogenic state of the DCs and results in the induction of Tregs. Crosstalk between C5aR and TLR2/4 or CR3 and TLR2/4 on APC surface results in diminished IL-12 production and downstream Th1 response. Finally, C1q and its receptors are crucial for the silent removal of apoptotic cells (i.e., provides pro-efferocytic signals) and this can influence T cell response.

While C3b opsonization is necessary to amplify complement activation via the AP, C3b plays a critical role in pathogen clearance. Erythrocytes bearing CR1 recognize and bind C3b-opsonized pathogens, and mediate transport of these complexes to the liver and spleen for clearance (Merle et al., 2015b). Transport may also be mediated by glycoprotein Ib (GPIb) on platelets, which captures C3b-opsonized pathogens and enhances adaptive immune responses via sDCs (Broadley et al., 2016). In the liver, the opsonized pathogens are carried to specialized Kupffer cells bearing CRIg, which can also bind C3b and mediate phagocytosis (Helmy et al., 2006). In the spleen, pathogens are transported to lymphoid follicles, which are protected by resident macrophages and DCs in the subcapsular sinus. Meanwhile, erythrocyte CR1, in the presence of FI, mediates cleavage of C3b to iC3b and/or C3d, which is recognized by CR2 and CR3. The subcapsular sinus macrophages and DCs can bind and internalize complement-tagged antigens and intact target cells, respectively, via CR3, and translocate them to naïve B cells or FDCs expressing CR2 in the lymphoid follicle (Merle et al., 2015b) (**Figure 2A**).

Within the lymphoid follicle, C3d mediates several functions that are critical mediators of B cell immunity, which have been reviewed previously in great detail (Carroll, 2004; Rickert, 2005; Bergmann-Leitner et al., 2006; Toapanta and Ross, 2006; Carroll and Isenman, 2012) (**Figures 2A,B**). Indeed, the roles of complement in the humoral antibody response have long been established. The first role involves acting as molecular adjuvant, effectively lowering the threshold for B cell activation (Carter et al., 1988) (**Figure 2B**). Crucial to C3d’s function in B cell mediated antibody response is its association with CR2. CR2 is part of the B cell co-receptor complex, together with CD19, CD81, and Leu13 (Matsumoto et al., 1991). Upon simultaneous engagement of both the BCR and the B cell co-receptor complex, the antigen threshold for B cell activation is reduced by up to four orders of magnitude (Carter and Fearon, 1992; Dempsey et al., 1996). In addition, BCR and B cell coreceptor crosslinking also enhances antigen processing and presentation to T cells (Cherukuri et al., 2001). Thus, C3d is especially critical for B cell stimulation in conditions where antigen concentration is low, as is typically the case during pathogen clearance (Carroll and Isenman, 2012). In addition to C3d’s direct role on B cells, it has been found that C3d-tagged immune complexes interact with FDCs to promote a more potent humoral response. Expression of CR1 and CR2 on FDCs promotes antigen reservoirs through binding and sequestering C3d-coated immune complexes in lymphoid follicles (Fischer, 1998). These immune complexes are cycled through non-degradative endosomal compartments within FDCs, which preserve the antigen for long periods of time (Heesters et al., 2013). These events promote the development of GCs, which is critical for maintenance of memory B cell repertoires (Fischer, 1998). Finally, C3d opsonization promotes extended half-life of antigens in blood, further facilitating generation of adaptive immune responses (Bergmann-Leitner et al., 2006).

Another mechanism of B cell regulation is mediated by C4BP. This regulator attaches to negatively charged moieties, including phospholipids of host membranes and other structures on bacterial surfaces (Blom et al., 2004). Beyond its complement inhibitory function, C4BP has been identified to specifically bind CD40, which is crucial for B cell activation and function. CD40, upon engagement with its nascent ligand CD40L, induces B cell proliferation, rescue of GC B cells from apoptosis, and CSR. Brodeur et al. (2003) found that C4BP by binding to CD40 could drive B cell activation, similar to what is seen by canonical CD40-CD40L interaction. A more recent study suggested that C4BP could also form a stable complex with soluble CD40L, and this complex upon association to CD40 provided signals that promoted cell survival without influencing proliferation (Williams et al., 2007). Unfortunately, the effect of this newly identified C4BP/CD40L complex was only observed for epithelial cell survival. Thus, future examination of these associations is required to fully elucidate the effects that C4BP has on lymphocyte biology and the adaptive immune response.

### Intrinsic T Cell Regulation by Complement

As a doctrine, complement has long been thought as a serum restricted system and its over 30 proteins could only be synthesized by the liver; however, this view is rapidly changing. It has now been identified that many disparate locations, and tissues in body have local sources of complement (for more detail discussion see, Kolev et al., 2014). Moreover, almost all cell types in the human body can produce complement proteins and many of those even contain intracellular complement stores (Liszewski et al., 2013).

Interestingly, both locally synthesized and intracellular complement have proven to be important for CD4+ T cell survival, proliferation, and differentiation (Heeger et al., 2005; Strainic et al., 2008; Liszewski et al., 2013; Kolev et al., 2015; Arbore et al., 2016). It was first identified by Heeger et al. (2005) that both naïve CD4+ T cells, as well as their cognate APC partner, could locally synthesize AP complement components C3, FB, and FD, and later it was shown C5 was also synthesized (Strainic et al., 2008). Local synthesis of these proteins was associated with a decrease in CD55, further enhancing AP activation (Heeger et al., 2005; Strainic et al., 2008). Interestingly, both the AT receptors C3aR and C5aR were found to be upregulated on both the T cell and APC during costimulation. Additionally, C3aR/C5aR activation on T cells provided signals to both induce IL-12 receptor expression and produce cytokines IL-12 and IFN-γ (Strainic et al., 2008) (**Figure 2C**). Given that IFN-γ supports the lineage commitment of naïve T cells to T helper 1 cells (Th1), this suggests that autocrine AT receptors are important in this process. In line with this notion, it was found that in the absence of autocrine T cell C3aR/C5aR signaling resulted in CD4+ T cells to commit to a Foxp3+ inducible regulatory T cell lineage (iTreg) (Strainic et al., 2012) (**Figure 2C**).

Excitingly, a new study linking autocrine AT receptor T cell signaling to another innate immune pathway was published. This study highlighted the role of C5aR in NLRP3 inflammasome activation, and found that these associations were tied to Th1 induction (Arbore et al., 2016) (**Figure 2C**). Upon TCR ligation and CD46 costimulation, intracellular C5 was cleaved and C5aR1 provided signals to drive NLRP3 inflammasome activation, which resulted in Th1 induction (Arbore et al., 2016). Surface-restricted C5aR2 was found to be a negative regulator of this process, promoting a regulatory T cell phenotype (**Figure 2C**). Thus, this study further strengthens the notion that autocrine C3aR/C5aR signaling is vital in skewing T cell differentiation and lineage commitment.

Many subsequent studies have further strengthened the role of complement on T cell survival, proliferation and differentiation. One study highlighted the potential importance of CD46 CTY-1 isoform in T cell regulation (**Figure 2C**). Coengagement of both TCR and CD46 in the presence of increasing IL-2 concentration induces IL-10 producing regulatory T cells (Cardone et al., 2010). Another recent study showed that intracellular C3 was cleaved into biologically active C3a and C3b by intracellularly expressed protease CTSL (Liszewski et al., 2013) (**Figure 2C**). Together these studies highlight the crucial role that local and intracellular complement plays in T cell biology, and opens the possibility that local complement is differentially regulated compared to serum complement.

Recently, studies have begun to unravel more unexpected roles for complement in driving fundamental T cell processes. Before activation, naïve CD4+ T cells are metabolically quiescent. Important for expansion and differentiation, these naïve cells must undergo major reprogramming such that they increase their nutrient uptake and usage of metabolic pathways. Interestingly, autocrine C3b stimulation of CD46, and specifically its intracellular domain CTY-1, was important for this metabolic change and for production of the proinflammatory cytokines needed for Th1 differentiation (Kolev et al., 2015) (**Figure 2C**). This study speaks to the fundamental nature that local complement plays on T cell biology. Remarkably, and in line with this notion, Notch family member Jagged1 was identified as the third physiologically relevant ligand for CD46 (Le Friec et al., 2012). The authors proposed that CD46 could sequester Jagged1 away from Notch to induce IFN-γ secreting Th1 cells. Altogether these aforementioned studies indicate that CRs (C3aR/C5aR, CD46) and their ligands are crucial regulatory components of T cell lineage commitment and survival.

### APC-Mediated Regulation (Paracrine)

Complement also plays an important role at the immunological synapse between APCs and T cells (**Figure 2D**). APCs (i.e., DCs) have been shown to express many of the same complement components as T cells, which also function in a similar fashion (Peng et al., 2008; Li et al., 2012). Extracellularly generated C3a/C5a was linked to increased expression of C3aR/C5aR on DCs, which increased both their activation [apparent by increased expression of costimulatory molecules (CD86 and MHCII)] as well as their costimulatory capacity (increased Th1 cytokine production) (Strainic et al., 2008; Li et al., 2012). Interestingly, blockade of the C3aR/C5aR signaling axis on DCs, such that extracellularly generated C3a/C5a could no longer bind, resulted in diminished costimulation capacity, apparent by a decrease in IFN-γ producing cells (Strainic et al., 2008). Independently, it was observed that using C5aR1 deficient DCs, but not C5L2, supported the induction of both Treg and Th17 lineages (**Figure 2D**). As in T cells, the two C5aR receptors and potentially C3aR seem to have independent roles in DCs (Weaver et al., 2010). These studies suggest that local complement generation by DCs provides important paracrine effects for T cell stimulation.

Beyond the AT receptor signaling axis, other complement regulators have also been shown to be important for DC differentiation/activation and downstream T cell response. Complement activation is under tight control by both membrane bound (CD46, CD55) and soluble (C4BP, FH) regulators. Two independent studies found that both FH and C4BP [β-chain lacking isoform(β-)] influence the early stages of monocyte to DC differentiation, and promote a tolerogenic/immature and anti-inflammatory DC phenotype (Olivar et al., 2013, 2016). Moreover, these DCs exhibited decreased production of proinflammatory Th1 cytokines, and increased production of IL-10 and TGF-β cytokines indicative of an anti-inflammatory response. Not so surprisingly, this resulted in impaired T cell proliferation and Th1 polarization, and instead induced a Foxp3+ Treg response (Olivar et al., 2016) (**Figure 2D**). Together, these studies suggest a non-redundant role of complement on DC differentiation and downstream T cell response, and that perhaps AP is not the only complement pathway governing T cell/DC differentiation and APC-T cell costimulation. To add to this notion, C1q has been found to have a unique function in antigen presentation and T cell priming. It is well-established that phagocytes not only internalize pathogens, but also ingest apoptotic host cells, a process that has been termed efferocytosis, both of which are influential on the cytokine response. Many studies have identified that C1q directly associates with both apoptotic DCs and macrophages (referred to C1q-polarized APCs) (**Figure 2D**). This association leads to increased efferocytosis and suppression of proinflammatory cytokine production (Korb and Ahearn, 1997; Baruah et al., 2009; Teh et al., 2011; Clarke et al., 2015). These dynamics led to suppressed Th1 and Th17 cell proliferation. These studies suggest a unique function of CP component C1q on APC and T cell costimulation, further supporting the notion that complement is truly a bridge between the adaptive and innate immune response.

### Other Mechanisms

#### C1q Receptors gC1qR and cC1qR Mediate APC and T Cell Stimulation

C1q is central to the activation of the CP of complement, and as aforementioned has unique function on modulating APC-driven cytokine production and downstream T cell priming. Structurally, individual C1q molecules are composed of a globular head situated on the carboxy termini of the collagen stock domain, and these molecules subsequently multimerize to form the prototypical hexameric “bouquet” superstructure (Sontheimer et al., 2005). Paradoxically, the functional relevance of the two co-expressed non-transmembrane cell surface associated receptors, gC1qR (interacts with globular head of C1q) and cC1qR (interacts with collagen stock of C1q), is still ambiguous (Frachet et al., 2015). However, while their exact function still remains controversial, studies suggest they may play a vital role in the adaptive immune response. We will now highlight our current understanding of these receptors and the potential role they play in driving the adaptive immune response.

C1q, as previously mentioned, is a crucial driver of pro-efferocytic signals and downstream T cell priming, although controversy still surrounds which of the C1qRs is important for this function (Nayak et al., 2012; Frachet et al., 2015). The prevailing hypothesis is that a tripartite interaction between C1q, gC1qR and cC1qR on apoptotic cells, and bystander phagocyte respectively, induces efferocytosis (Hosszu et al., 2012; Frachet et al., 2015) (**Figure 2D**). Recently it was shown that gC1qR, which is present on both immature DCs and blood precursor DCs, interacts with DC-SIGN (Hosszu et al., 2012). As both C1qRs lack transmembrane domains and cannot signal on their own and must recruit signaling partners, DC-SIGN is now postulated to be a transmembrane signaling partner of gC1qR. Interestingly, authors suggested that DC-SIGN also directly interacts with C1q, and given C1q’s role on DC differentiation, postulated that perhaps this trimolecular receptor complex between C1q-gC1qR-DC-SIGN might also influence DC differentiation (Hosszu et al., 2012). Additionally, gC1qR can also directly influence cytokine response. Crosstalk between gC1qR and TLR4 resulted in a dampened IL-12 response, a cytokine important for IFN-γ production and subsequently Th1 cell proliferation and differentiation (Waggoner et al., 2005). Furthermore, C1q can directly bind to T cells via C1qRs and induce anti-proliferative effects (Chen et al., 1994). It has been suggested that gC1qR is pivotal in the regulation of the antiviral T cell response. Another study found that both of the C1qR’s offered a unique function on the differentiation process from monocytes to DCs. Immature monocytes were found to have high levels of gC1qR and low levels of cC1qR, a phenotype that was reversed as the cells began to differentiation to a DC lineage. The authors theorized that C1q/C1qR system was crucial for the regulation and transition from innate monocyte state to that of a professional APC (Hosszu et al., 2010). Thus, we are still trying to understand the role that gC1qR plays in driving acquired immunity.

#### Crosstalk between Complement and Toll-Like Receptors

Toll-like receptors and CRs are both critical to the innate immune response and are co-expressed on leukocytes. Thus, it is not surprising that crosstalk between these classes of receptors has been demonstrated. Recent evidence supports a role of CR-TLR crosstalk in instructing adaptive immune response (Hajishengallis and Lambris, 2010, 2016). Mechanistically, this crosstalk has largely been observed to be at the MAPK level, specifically through the ERK1/ERK2 pathway (Hajishengallis and Lambris, 2010).

It is becoming increasingly clear that the AT receptors have a multifaceted role in guiding the host immune response, that reaches beyond its canonical function in complement. As mentioned above, these receptors are crucial in antigen presentation and T cell survival/response. Interestingly, the AT receptors have also been found to influence TLR driven cytokine production and downstream T cell responses. In particular, C5aR, depending on the immunological cell, has been shown to have paradoxical function on TLR cytokine response. On DCs, it was identified that C5aR-TLR2 crosstalk could synergistically enhance TLR driven IL-12 production, which resulted in an increased Th1 response (Weaver et al., 2010). Conversely, other studies have provided evidence that C5aR-TLR2 and C5aR-TLR4 crosstalk on macrophages inhibit IL-12 family of cytokine, specifically IL-12p70, thereby diminishing the Th1 response (**Figure 2D**). Thus, the immunological consequence of AT receptor-TLR crosstalk seems largely dependent on the type of APC.

Crosstalk between CR3 and TLRs has also been identified, which also has an influence on TLR cytokine response and quality of the adaptive immune response. Much like what was seen with C5aR, CR3 when co-activated with either TLR2 or 4, leads to suppressed IL-12 (IL-12p70) expression and accordingly a diminished Th1 response (**Figure 2D**). However unlike C5aR, TLRs are able to activate CR3 through a cellular process that has been termed “inside-out” signaling. Although using slightly different pathways, this has been verified for both TLR2-CR3 and TLR4-CR3 activation. CR3-TLR2 activation seems to be dependent on PI3K, meanwhile TLR4-driven CR3 activation is driven by MyD88. Engagement of CR3 alone by iC3b-coated cells has also been shown to downregulate IL-12 production (Marth and Kelsall, 1997). Additionally, gC1qR-TLR crosstalk has also been demonstrated but has redundant effects to C5aR/CR3-TLR2, -TLR4 on IL-12 expression. Taken together, these findings suggest yet another strong tie between complement and adaptive immunity.

## Complement Evasion by Pathogens

While complement offers a potent immunological barrier, pathogenic organisms have developed counterstrategies to elude harmful responses. Pathogenic complement evasion has been extensively studied, and countless mechanisms and examples are described in literature (reviewed in Lambris et al., 2008; Stoermer and Morrison, 2011; Ricklin, 2012; Zipfel et al., 2013; Merle et al., 2015b; Garcia et al., 2016). These mechanisms include secretion of complement inhibitory molecules, recruitment of host complement regulators, and proteolytic cleavage of complement effector molecules. Most examples of complement evasion have been studied in the context of innate immune blockade, however given the close ties between complement and adaptive immunity, it is likely that these mechanisms also modulate B and T cell responses. In the remainder of this review, we highlight specific examples of pathogenic evasion of complement-mediated adaptive immunity (**Table 1**; **Figure 1**). Based on these examples, we also postulate possible new roles for known evasion molecules.

**Table 1:** Mechanisms for pathogenic evasion and modulation of complement-mediated adaptive immunity.

Organism	Evasion molecule	Mechanism	Function(s)
**Blocking B cell immunity**			
*S. aureus*	Efb, Ecb, Sbi	Binds C3d, inhibits CR2 binding	Inhibits B cell stimulation
*P. berghei*	CSP	Binds C3d, likely inhibits CR2 binding	Prevents antibody production
*C. albicans*	MP60	Binds C3d	Enhances cell adherence
HSV-1	gC-1	Binds C3b, prevents complement activation	Reduces viral antibody titers (C3 and CR1/2 necessary for adaptive response)
HCV	NS5A, HCV core	Inhibits C3 and C4 mRNA transcription, upregulates CD55	Inhibits binding to B cells (C3 and CR1/2 necessary for adaptive response)
HIV	gp41	Binds FH, enhances surface iC3b/C3d deposition, attaches to CR2-bearing cells	Generates viral reservoirs, enhances cell infectivity
*S. aureus*	SCIN	Binds C3b, inhibits CR1 and CRIg binding	May block CR1-mediated trafficking and B cell binding/stimulation^1^
**Blocking direct T cell regulation**			
HCV	HCV core	Binds gC1qR (on T cells)	Decreases IL-2 and IFN-γ, inhibits Th1 and promotes Th2 response
HIV	gp41	Binds gC1qR (on T cells)	Induces NKp44L expression, facilitates NK-mediated killing of uninfected T cells
*S. pyogenes*	M1	Binds CD46 (on T cells)	Induces IL-10 secreting Tregs
Measles virus	Hemagglutinin	Binds CD46 (on monocytes and DCs)	Inhibits IL-12 production, enhances IL-10 production, but may enhance inflammatory response (in some cases)
*M. leprae*	PGL-1	Binds C3, mediates binding to CD46 (on T cells)	Induces IL-10 secreting Tregs
*S. aureus*	SpA	Binds gC1qR	May inhibit Th1 and promote Th2 response^1^
*L. monocytogenes*	InlB	Binds gC1qR	May inhibit Th1 and promote Th2 response^1^
**Blocking indirect T cell regulation**			
*P. gingivalis*	HRgpA, RgpB	Cleaves C5, C5a binds C5aR (on macrophages)	Manipulates C5aR-TLR2 crosstalk, inhibits IL-12 and IFN-γ production, inhibits Th1 response
*P. gingivalis*	Fimbrae proteins	Binds CR3	Manipulates CR3-TLR2 crosstalk, inhibits IL-12 production, enhances pathogen clearance
*L. major*	gp63	Cleaves C3b to iC3b, facilitates CR3 binding	Inhibits IL-12 production, enhances IL-10 production
*B. anthracis*	BclA	Binds CD14 and CR3	Manipulates CR3-TLR2 crosstalk for cell entry
*F. tularensis*	?	Binds CR3	Inhibits TLR2-mediated proinflammatory cytokine production
*B. pertussis*	FHA	Binds CR3	Inhibits IL-12 production
HIV	gp41	Binds FH and enhances surface iC3b, or can bind CR3 directly	Manipulates CR3/TLR8 crosstalk, inhibits antiviral and proinflammatory responses
*S. aureus*	CHIPS	Binds and inhibits C5aR	May manipulate C5aR-TLR2 crosstalk, inhibit IL-12 production and Th1 response^1^
**Other inhibition mechanisms**			
Vaccinia virus	VCP	Cleaves C3b to iC3b	Inhibits CD4+ and CD8+ T cell response (dependent on C3)
WNV	NS1	Binds FH, mediates C3b cleavage Binds C4BP, mediates C4b cleavage Binds C1s to cleave C4	Inhibits CD4+ and CD8+ T cell response (C3 and CR1/2 necessary for adaptive response)

*^1^The functional outcome of these evasion mechanisms has not been definitively shown, but is postulated based on the results from other known mechanisms.*

### Blocking B Cell Immunity

C3d is the smallest of the C3 fragments that opsonizes antigens and target cells, and plays a number of critical roles in directing adaptive immunity, as described above. Interestingly, C3d is a “molecular hub” of sorts, binding not only host complement regulators and receptors, but also a multitude of immune evasion molecules. While many of these molecules have been studied extensively, most work focuses on their role in inhibition of C3 and C5 cleavage, and resulting direct complement inhibitory functions. However, some studies show that blockade of C3d can lead to impaired adaptive immune responses.

*Staphylococcus aureus* is a Gram-positive bacterium that colonizes a large proportion of the human population. However, this bacterium can also become pathogenic, and is responsible for severe infections. *S. aureus* is considered a master of immune evasion. Indeed, tens of virulence factors have been identified, which influence innate immunity through inhibition of neutrophil chemotaxis and intracellular killing mechanisms, phagocytosis, complement, TLR signaling, and also by directly killing host cells. *S. aureus* also modulates adaptive immune responses directly, using superantigens that crosslink TCRs and MHCII molecules and hyperactivate T cell responses (Thammavongsa et al., 2015; Koymans et al., 2016). In particular, complement evasion is arguably best understood in the case of *S. aureus*, in which numerous evasion molecules exert control over several points in the complement cascade. Despite their well-characterized roles in complement evasion, it remains unclear whether most of these molecules influence complement-mediated adaptive immunity. Notably, two homologous virulence factors from *S. aureus*, Efb-C and Ecb (also known as Ehp) influence B cell immunity. Initially, these molecules were described to bind nearly all thioester-containing C3 proteins and inhibit the complement AP (Lee et al., 2004; Hammel et al., 2007a,b; Jongerius et al., 2007). A follow-up study demonstrated that both proteins can also inhibit the interaction between C3d and CR2, and prevent B cell stimulation, which may act as another evasion strategy for *S. aureus* (Ricklin et al., 2008). Additionally, there is a case for Sbi having a similar role, as it also competitively inhibits the C3d-CR2 interaction (Burman et al., 2008; Isenman et al., 2010).

Other pathogens produce proteins that bind C3d as well. The CSP from *Plasmodium berghei* can bind to C3d, preventing production of antibodies against the antigen and reducing protective immunity during associated malaria infection (Bergmann-Leitner et al., 2005). The mechanism of modulating antibody response was not elucidated, but it is presumed that CSP also blocks the C3d-CR2 interaction, which interferes with stimulation of B cells in the lymphoid follicle. *Candida albicans* expresses MP60 and mannoproteins that bind C3d and mediate binding to host cells (López-Ribot et al., 1995; Stringaro et al., 1998), however, the functional consequences on adaptive immunity remain unclear.

Some pathogens interfere with complement-mediated B cell immunity through modulating events in the complement cascade upstream of C3 deposition. HSV-1 expresses a glycoprotein (gC-1), which binds to C3b and blocks binding sites for C5 and properdin (Kostavasili et al., 1997). In addition, gC-1 can mediate decay of C3 convertase enzymes, limiting the amount of complement opsonization on the viral surface (Fries et al., 1986). While this protein is described to inhibit complement-mediated neutralization of HSV-1 (Lubinski et al., 2002), another study with C3 and CR1/2 deficient mice showed reduced IgG response to the virus, suggesting that complement is critical in development of adaptive immune response against HSV-1 (IgG and GCs) (Da Costa et al., 1999). Thus, gC provides an important evasion strategy for HSV-1 to escape complement-driven adaptive immunity. HCV influences complement activation via distinct mechanisms. The HCV core protein and NS5A target USF-1 and IRF-1, leading to inhibited mRNA transcription in infected hepatocytes, and diminished C4 levels in mice (Banerjee et al., 2011). A follow-up study showed that NS5A also inhibits C3 mRNA transcription. Indeed, human patients with HCV showed low C3 levels in serum, and biopsies showed lower C3 mRNA levels in the liver (Mazumdar et al., 2012). Another recent study showed that HCV core protein induced soluble CD55 expression, which limits complement activation in infected hepatocytes (Kwon et al., 2016). Each of these mechanisms can inhibit viral opsonization and in turn may inhibit complement-mediated B and T cell responses. Finally, Wang et al. (2016) demonstrated that HCV binds to B cells via interactions between C3 fragments and either CR1 or CR2. Thus, by inhibiting opsonization, HCV could escape recognition by B cells. Another possible mechanism was proposed for SCIN, which mediates dimerization of C3b molecules and AP C3 convertases on the bacterial surface. In doing so, SCIN effectively masks the binding sites for CR1 and CRIg, preventing phagocytic responses and cleavage of C3b to iC3b (Jongerius et al., 2010). In turn, SCIN likely inhibits CR-mediated immune adherence and trafficking, B cell stimulation, and antigen presentation.

In contrast to C3d-CR2 blockade, viruses can exploit this interaction to promote their survival and pathogenesis. HIV, for example, carefully balances complement activation and regulation in order to capitalize on the links between complement and B cell immunity. While HIV virions indeed activate complement (Sullivan et al., 1998), the virus incorporates membrane-bound complement regulators from the infected host cell, including CD46, CD55, and CD59 (Montefiori et al., 1994). Additionally, HIV glycoproteins gp41 and gp120 recruit FH to the viral surface (Stoiber et al., 1995). These regulators limit the amount of C3 deposition on the virus and prevent direct lysis by MAC. During infection, HIV disseminates through the bloodstream, becomes opsonized with C3b, and encounters erythrocytes bearing CR1 (Horakova et al., 2004). These erythrocytes then bind and transport complement-opsonized virions to secondary lymphoid organs (Schifferli et al., 1988). Meanwhile, CR1 can also act as a cofactor for FI-mediated cleavage of C3b to iC3b and eventually C3d, which facilitates transfer of HIV from erythrocytes to B cells and FDCs bearing CR2. HIV leverages these interactions to promote maintenance of extracellular viral reservoirs in the lymphoid follicle, where virions are captured on CR2-expressing FDCs (Bergmann-Leitner et al., 2006). Several lines of evidence demonstrate that intact HIV virions can be maintained for over 6 months in GCs (Cavert et al., 1997). Although these virions are “trapped” and opsonized, they still retain infectivity once released (Banki et al., 2005). Most importantly, the high concentration of HIV in the lymphoid follicle promotes infection of CD4+ T cells, which is critical for the pathogenesis of the virus (Stoiber et al., 2008).

### Inhibiting T Cell Immunity through CR Engagement

CRs play a critical role in T cell proliferation and differentiation, as described in detail above. Thus, many pathogens have also evolved strategies to engage these receptors in order to modulate T cell immunity in their favor.

As described above, HCV produces multiple proteins that modulate levels of complement proteins in infected hepatocytes. In particular, the HCV core protein inhibits C3 mRNA transcription, while upregulating soluble CD55 production. Another important role of HCV core protein includes inhibition of T cell proliferation through its interaction with gC1qR on DCs, B cells, and T cells (Kittlesen et al., 2000). Binding of HCV to gC1qR on activated T cells decreases IL-2 and IFN-γ production (Yao et al., 2001a). Engagement of gC1qR on DCs inhibited IL-12 production, and promoted a shift from Th1 to Th2 response (Waggoner et al., 2007). A later study showed that patients with chronic HCV infection had persistent elevated levels of gC1qR+ CD4+ T cells, which increased the susceptibility to viral evasion via HCV core protein (Cummings et al., 2009). Other viruses, including HIV, EBV, and HSV, are also known to engage gC1qR, and may induce similar effects on T cell immunity (Yao et al., 2001b; Fausther-Bovendo et al., 2010). Similarly to HCV, HIV can also engage gC1qR. HIV expresses glycoprotein gp41 on its surface, which during fusion with the membrane of infected CD4+ T cells, can interact with gC1qR on uninfected T cells. This interaction induces expression of NKp44L, a ligand for cytotoxicity receptor NKp44 on NK cells. This mechanism causes NK cells to selectively deplete uninfected CD4+ T cells during HIV pathogenesis (Fausther-Bovendo et al., 2010).

Numerous other pathogens produce molecules that bind gC1qR, which may enable them to subvert undesired T cell responses. For instance, *S. aureus* produces SpA, a protein known to bind many ligands and contributing to the virulence of numerous strains. Perhaps the most well-known function involves binding of IgG molecules via their Fc region, in order to avoid bacterial opsonization and subsequent phagocytosis. SpA was also reported to bind gC1qR on platelets, and while the exact physiological role of this interaction is unclear, it may be involved in adherence of *S. aureus* to sites of vascular injury (Nguyen et al., 2000). Based on the aforementioned evasion mechanisms, it is plausible that *S. aureus* uses SpA to bind gC1qR on T cells, in order to downregulate IL-12 and prevent Th1 response. In addition, *Listeria monocytogenes*, another infectious bacterium, expresses another gC1qR-binding protein called InlB. The bacterium utilizes InlB to enter gC1qR-expressing mammalian cells, facilitating intracellular uptake and survival of the bacterium. InlB may allow specific entry of *L. monocytogenes* into CD4+ T cells expressing gC1qR as well, simultaneously escaping aggressive Th1 response (Braun et al., 2000).

Aside from gC1qR, CD46 is another CR present on T cells, which plays an important role in modulating T cell response. Group A Streptococcus (*Streptococcus pyogenes*), a Gram-positive bacterial pathogen, comprises numerous immune evasion mechanisms, similarly to *S. aureus*. *S. pyogenes* strains are characterized by the expression of distinct M proteins that protrude from and mask the bacterial surface. Different M serotypes bind a wide variety of host proteins and confer bacterial resistance to immune responses. A recent study showed that several M serotypes can bind directly to CD46 on humans CD4+ T cells, resulting in the induction of IL-10 secreting regulatory T cells upon costimulation with an anti-CD3 antibody. These data suggest that M protein, by exploiting the immunomodulatory function of CD46, could delay effector T cell response and allow *S. pyogenes* to further establish infection (Price et al., 2005). Several additional bacteria and viruses, including Measles virus, HHV-6, *Escherichia coli*, and *Neisseria* bind CD46 and influence cytokine profiles of APCs and T cells, but it remains unclear whether these pathogens engage and modulate T cells directly (Cattaneo, 2004; Kemper and Atkinson, 2009). Measles virus hemagglutinin is known to bind CD46, which results in IL-12 downregulation in primary monocytes and DCs (Karp et al., 1996; Marie et al., 2001). In a later study, transgenic mice expressing either of the two human CD46 molecules (with differing cytoplasmic tails) were injected with vesicular stomatitis virus expressing measles virus hemagglutinin. Mice expressing CD46-1 showed enhanced IL-10 production and immunosuppressive response, while those expressing CD46-2 exhibited enhanced inflammatory response (Marie et al., 2002). These results are further complicated with the observation that Measles virus downregulates CD46 on T cells *in vivo*, and preferentially engages an alternate receptor (Yanagi et al., 2006). Thus, the role of CD46 in Measles virus pathogenesis remains somewhat unclear.

*Mycobacterium leprae*, the pathogen responsible for causing leprosy, engages CD46 using a slightly different mechanism. It has long been known that *M. leprae* negatively affected DC maturation and downstream T cell response, but how this occurred remained elusive due to lack of appropriate models. However, using non-virulent *M. bovis* BCG, it was identified that PGL-1, which had been suspected to influence the pathogenesis of *M. leprae*, was the culprit. In fact, PGL-1 was able to exploit CR3 to allow entry of BCG into DCs, which subsequently led to dampened DC maturation (Tabouret et al., 2010). A parallel study found that PGL-1 was exposed on surface of infected DCs and lipid rafts of T cells, where it was able to bind C3. Interestingly, CD46 on T cells was able to recognize this C3-PGL-1 complex on *M. leprae* infected human DCs resulting in the differentiation of IL-10 producing Tregs (Callegaro-Filho et al., 2010). Together these studies provide evidence that mycobacterium, by associating with various complement components, can directly affect the outcome of the adaptive immune response. In addition, a new study, found that FH plays a defining role in the internalization process of *M. bovis* BCG in macrophages. The authors found that FH, by directly binding to the *M. bovis* BCG, could affect the uptake of *M. bovis* BCG and thereby alter the cytokine response. While it remains unclear FH binding might affect the outcome of the adaptive immune response, it is clear mycobacterium affects multiple parts of the complement cascade, leading to altered T cell response (Abdul-Aziz et al., 2016).

In addition to the mechanisms outlined, CD46-mediated T cell regulation could be a general evasion mechanism for pathogens. Upon complement activation, pathogens become opsonized by C3b, which can bind CD46 on T cells. In combination with CD3 costimulation, this event could help drive IL-10 induction and regulatory T cell response against these microbes. Furthermore, complement-opsonized pathogens that are internalized by T cells could achieve a similar response by engaging intracellular CD46.

### Indirectly Modulating T Cell Immunity via APCs

In the last decade, numerous links between complement activation and TLR signaling have been uncovered, which can drive adaptive immune responses (Hajishengallis and Lambris, 2010). Likewise, pathogens have developed strategies to evade these immune mechanisms (Hajishengallis and Lambris, 2011).

*Porphyromonas gingivalis* is a Gram-negative bacterium that is known to cause dysbiosis within the periodontal microbiome. This bacterium is a prime example of how exploitation of CR-TLR crosstalk can direct adaptive immunity. *P. gingivalis* produces two enzymes, HRgpA and RgpB, which directly cleave C5 into C5a and C5b. The resulting C5a can then engage C5aR1, while bacterium simultaneously binds TLR2 (Wang et al., 2010), and the C5aR1-TLR2 crosstalk can influence cytokine profiles through different signaling cascades. Interestingly, the functional role of these interactions is entirely different depending on the cell type involved. In macrophages, C5aR1-TLR2 crosstalk can selectively inhibit IL-12 and IFN-γ production, through induction of PI3K and ERK1/2 signaling. This evasion mechanism allows the pathogen to induce a customized adaptive immune response, preventing Th1 induction (Liang et al., 2011). At the same time, this signaling leads to increased cAMP production and leads to downregulation of antimicrobial nitric oxide production. However, in neutrophils, C5aR1-TLR2 signaling mediates proteosomal degradation of MyD88 and activation of an alternate signaling pathway, which inhibits phagocytosis, but maintains inflammatory responses to propagate periodontal dysbiosis (Maekawa et al., 2014). In contrast, C5aR1 signaling mediates bacterial killing in DCs. This evidence indicates that the pathogen has tailored leukocyte evasion to its environment, where it primarily encounters neutrophils and macrophages, but not DCs (Hajishengallis and Lambris, 2016). The mechanisms underlying the variable signaling are still unclear.

*Porphyromonas gingivalis* also utilizes an additional mechanism to influence IL-12 production via CR-TLR crosstalk. The fimbrial proteins, expressed on the bacterial surface, engage CR3 and mediate internalization and intracellular survival within macrophages, as well as reduced IL-12 production. This mechanism was shown to be dependent on TLR2, which also binds fimbrial proteins and induces inside-out signaling that activates CR3 (Wang et al., 2007). Experiments in mice showed that blockade of CR3 restored IL-12-mediated pathogen clearance, thus demonstrating that this immune mechanism also promotes pathogen survival and escape of adaptive immune response (Hajishengallis et al., 2007).

In addition, numerous other pathogens can bind both CRs and TLRs to infect host cells, inhibit IL-12 production, and direct T cell immunity (Hajishengallis and Lambris, 2011, 2016). *Bacillus anthracis* spores infect professional phagocytes in their host to promote their growth and survival. The outer layer of *B. anthracis* spores contain a glycoprotein, BclA, which mediates cell internalization via CR3 (Oliva et al., 2008). Since CR3 must be activated in order to facilitate internalization, it was unclear how BclA leveraged this receptor to invade host cells. Interestingly, a subsequent study found that BclA also binds CD14, which induces TLR2-mediated inside-out signaling to activate CR3 and internalize *B. anthracis* spores (Oliva et al., 2009). *Francisella tularensis* strain Schu S4 also subverts immune response via CR3 and TLR2. C3-opsonized *F. tularensis* is internalized by macrophages via CR3, and through outside-in signaling mechanisms, blocks TLR2-mediated proinflammatory cytokine production (Dai et al., 2013). *Bordetella pertussis* FHA inhibits IL-12 production and binds CR3, but it remains unclear whether these events are connected (Ishibashi et al., 1994; McGuirk and Mills, 2000). Another interesting CR-TLR evasion mechanism was recently uncovered for HIV, in which the complement-opsonized virus engages both CR3 and TLR8 outside and within DCs, respectively. The CR3-TLR8 signaling crosstalk led to reduced antiviral and inflammatory cascades, while promoting viral transcription and replication (Ellegård et al., 2014). Whether or not these CR-TLR crosstalk events direct T cell responses requires further investigation.

It is possible that coengagement of CRs and TLRs represents a general strategy of immune modulation, since most opsonized microbes are likely to engage both simultaneously. The question remains as to whether these events trigger pathways involved in clearance or killing, or whether they promote pathogenic survival. In the case of *P. gingivalis*, for example, C5aR1-TLR2 coengagement can induce many different immunological outcomes depending on cell type. Thus, further investigation of these signaling mechanisms may be critical for understanding how pathogens exploit CR-TLR crosstalk. One related evasion strategy was recently proposed for *S. aureus*. As described above, activation of both TLR2 and C5aR1 drives Th1 response in splenic DCs. *S. aureus* expresses both TLR2 inhibitors (i.e., SSL3) and C5a inhibitors (i.e., CHIPS), which can inhibit C5aR signaling in sDCs, and thereby shift T cell response toward Th17 (Weaver et al., 2010).

It has also been established that microbes use CR3 to promote immunologically silent entry into host cells (Marth and Kelsall, 1997). Many pathogens cleave C3b to iC3b on their surface, which promotes binding to CR3. This is achieved through recruitment of host complement regulators, host proteases, or secretion of endogenously expressed proteases (Potempa and Potempa, 2012). Some strains of the eukaryotic parasite Leishmania produce a glycoprotein (gp63) on the surface of the parasite that can cleave C3b into inactive form iC3b, resulting in the inhibition of convertase formation and terminal complement activation (Brittingham et al., 1995). Allowing iC3b to associate with CR3 on APCs inhibits IL-12 production (Marth and Kelsall, 1997), and although not directly shown, results in altered T cell response (Da Silva et al., 1989). As described above, HIV also exploits CR3 to infect immune cells while avoiding harmful inflammatory cascades. In order to improve its chances at CR3 recognition, HIV glycoproteins gp120 and gp41 can recruit FH and mediate C3b breakdown to iC3b, and gp41 may even engage CR3 directly (Stoiber et al., 1995).

### Other Mechanisms of Complement Inhibition

There are several examples in which pathogens inhibit complement activation and influence T cell immunity, through mechanisms that are not fully understood. Poxviruses are a large family of viruses (69 members) including smallpox virus and vaccinia virus, which employ novel strategies to evade complement-mediated recognition and clearance. These viruses express complement regulator mimics, which are comprised of up to four CCP domains. These proteins bind C3b and C4b in a similar manner to host complement regulatory proteins, promoting convertase decay and opsonin cleavage (Ojha et al., 2014). VCP from vaccinia virus is one example that, in addition to its direct complement regulatory role, can inhibit CD4+ and CD8+ T cell responses. In an intradermal infection model, mice infected with a VCP-knockout strain of vaccinia virus had increased numbers of CD4+ and CD8+ T cells at the site of infection, and exhibited increased T-dependent antibody response, compared to infection with the wild-type virus. Interestingly, no difference in T cell responses was observed in C3^-/-^ mice, indicating that the role of VCP in inhibition of T cell responses is complement dependent. The authors of this study suggest that functionally homologous molecules from other poxviruses may play a similar role in viral evasion of T cell immunity (Girgis et al., 2011).

Poxviruses are not the only family of virus that evades complement mediated adaptive response; flaviviruses do as well. Flaviviruses are positive stranded RNA viruses, and include viruses that to this day can be deadly, such as dengue virus. One strong example of the mechanisms used by flavivirues to evade the host immune defense comes from WNV. Adaptive immune response against WNV is dependent on all three complement pathways. In particular, the AP drives CD4+ and CD8+ T cell responses directly, without affecting antibody titers, as demonstrated by FB^-/-^ mice (Mehlhop and Diamond, 2006). NS1 from WNV, in similarity to NS1 from other flaviviruses, binds FH and facilitates FI-mediated cleavage of C3b (Chung et al., 2006). Subsequent studies showed that NS1 can form a complex with both C1s and C4, causing rapid fluid-phase consumption of C4 and preventing C4b deposition on the viral surface (Avirutnan et al., 2010). Finally, NS1 can also bind C4BP and promote cleavage of C4b, blocking C3 activation via the CP and LP (Avirutnan et al., 2011). Additionally, mice lacking C3 or CR1/CR2 genes exhibited suppressed humoral immunity against WNV (Mehlhop et al., 2005). This multifaceted blockade of the complement cascade not only prevents direct complement-mediated viral clearance, but would likely also inhibit T cell immunity critical for viral clearance, since these responses are dependent on C3 and the AP (Suthar et al., 2013). Although direct evidence has only been shown for WNV, it is possible that NS1 from other flaviviruses use a similar mechanism to evade complement mediated adaptive immune response. Thus, future studies should focus on the role of NS1 from other deadly flaviviruses in directing the host immune defense.

Other viruses secrete molecules that directly block complement activation, which may dampen adaptive immune responses. For example, the M1 protein from influenza virus blocks the interaction between IgG and C1q, thereby inhibiting CP activation of complement (Zhang et al., 2009). Independent studies showed that blockade of C5aR in an influenza infection model abrogated CD8+ T cell response (Kim et al., 2004). Furthermore, mice deficient in C3 exhibited diminished migration of CD4+ and CD8+ T cells during influenza infection, resulting in delayed viral clearance and increased viral titers. CR1/CR2 deficiency had no effect on this phenomenon, indicating that it is not driven by complement-mediated B cell stimulation (Kopf et al., 2002). Thus, M1, by inhibiting CP complement activation, can prevent both complement-mediated and T cell-mediated clearance of influenza virus.

A recent groundbreaking study showed an intracellular role of C3, independent of complement activation. After pathogens tagged with C3 extracellularly (via the canonical complement activation cascades) are internalized, C3 products are detected, and triggers NF-κB, IRF-3/5/7, and AP-1 signaling cascades, leading to the release of proinflammatory cytokines, and proteosomal degradation of infecting viruses (Tam et al., 2014). Certain viruses (i.e., rhinovirus and poliovirus) encode proteases that can degrade opsonizing C3 fragments, promoting their intracellular survival.

Overall, it is likely that complement inhibition or modulation on any level will influence adaptive immune responses against pathogenic organisms. We have described numerous mechanisms by which regulating opsonization, promoting complement cleavage, interfering with CR signaling, and direct engagement of CRs can alter B and T cell responses. Further studies are required to understand the roles of the diverse array of immune evasion molecules on the adaptive fitness of their respective organisms.

## Perspectives

Although complement was once regarded as a self-contained effector arm of innate immunity, its collaboration with other immunological phenomena is continually emerging. Indeed, complement has been long known to bridge innate and adaptive immunity through the C3d-CR2 interaction, mediating B cell activation, antigen presentation, and generation of immunological memory. More recently, complement has been implicated in driving T cell immunity, both through modulating cytokine profiles in APCs, as well as through direct engagement with receptors on and inside T cells. While our view of complement has expanded dramatically, we have likely just scratched the surface. In the era of crosstalk between the different branches of immunity and beyond, many additional roles of complement are waiting to be discovered.

Complement evasion is well-documented among microorganisms. Indeed, many immune evasion molecules have been discovered, and their mechanisms of complement modulation are now clearly understood. In light of the numerous links between complement and adaptive immunity, the question arises as to whether complement evasion molecules modulate B and T cell responses. In general, there is a relative lack of studies describing how pathogens exploit complement-mediated adaptive immunity. This is due, in large part, to the infancy of the field. Many of these phenomena were discovered within the last 5–10 years. Additionally, the complexity of these immune processes makes them difficult to study. Although some complement evasion molecules are linked to altered T cell responses, determining the underlying molecular mechanisms is challenging. Recent advancement in various sequencing, mouse, and cytometric technologies affords the opportunity to address these more complex and fundamental questions regarding complement and T cell biology. However, the species specificity of many immune evasion molecules makes it difficult to study their effects in mouse models. Finally, there is the notion that bacterial immunity is primarily handled by innate effector functions. Indeed, while viral clearance is primarily T cell mediated, bacteria are recognized in the extracellular milieu by pattern recognition molecules and receptors, which mount a rapid and sometimes aggressive innate immune response against the invading pathogen. These responses often also promote adaptive immune responses and the generation of immunological memory, but historically these responses are considered secondary to innate immunity. However, antibodies are often essential for efficient complement activation on bacteria. Accordingly, while bacteria have huge arsenals of virulence factors targeting innate immune components (including complement), direct evasion of adaptive immunity seems less prevalent, though it does exist (i.e., superantigens of *S. aureus*). With the continuing discovery of diverse roles of complement in directing adaptive immunity, and new tools available to study interactions of complement and leukocytes, it is likely that many bacterial complement inhibitors may block B and T cell responses.

Despite its important role in controlling microbial infections, complement is also implicated in numerous autoimmune and inflammatory conditions. Excessive and uncontrolled complement activation plays a role in many diseases, either directly (i.e., aHUS, PNH) or indirectly (i.e., RA, SLE, organ transplantation) (Ricklin and Lambris, 2013; Morgan and Harris, 2015). This activation can also drive adverse adaptive immune responses. In SLE, for example, complement activation on damaged or apoptotic cells may lead to generation of autoreactive antibodies promoted by C3d-CR2 B cell stimulation (Holers, 2014). Additionally, recent evidence has shown that complement drives both B and T cell responses during transplantation (Sacks and Zhou, 2012). Thus, microbial evasion molecules may hold promise for directing complement-mediated adaptive immune responses for treatment of a new array of disease, which were previously considered not to be amenable to complement modulatory strategies. Conversely, the power of complement can be harnessed to promote favorable adaptive immune responses. Since the discovery of its adjuvant potential, C3d has been exploited for development of more potent vaccines (Toapanta and Ross, 2006). Furthermore, there are possibilities to leverage known mechanisms of complement-mediated T cell immunity for treatment of infectious diseases and cancer.

## Conclusion

The physiological role of complement has greatly expanded in recent years. It is now accepted that complement is a crucial mediator of adaptive immune responses. In addition to its long-known role in regulating B cell immunity via C3d-CR2, more recent work has established a multifaceted approach by which complement drives T cell responses. These mechanisms include direct engagement between complement activation products with CRs on T cells, indirect regulation through APC engagement, and modulation of cytokine profiles through CR-TLR crosstalk. It is only natural that pathogens, in their struggle for survival, have developed strategies to overcome these immune pathways. While numerous evasion mechanisms of complement-mediated adaptive immunity have been characterized, it is likely that many other evasion strategies remain undiscovered, including those mediated by known immune evasion molecules. A better understanding of pathogenic modulation of complement-mediated adaptive immunity is prerequisite to capitalizing on the therapeutic potential of immune evasion molecules.

## Author Contributions

KB, SR, and RG conceived the concept for this review article. KB and RG wrote the manuscript. KB, SR, and RG read, edited, and reviewed the manuscript.

## Conflict of Interest Statement

The authors declare that the research was conducted in the absence of any commercial or financial relationships that could be construed as a potential conflict of interest.

## Abbreviations

aHUS: atypical hemolytic uremic syndrome
AP: alternative pathway
APC: antigen presenting cell
AT: anaphylotoxin
*B. anthracis*: Bacillus anthracis
B. pertussis: *Bordetella pertussis*
BclA: *Bacillus* collagen-like protein of anthracis
BCR: B cell receptor
C4BP: C4b-binding protein
cC1qR: collagen C1q receptor
CD46: membrane cofactor protein
CD55: decay accelerating factor
CCP: complement control protein
CP: classical pathway
CR: complement receptor
CRIg: complement receptor of immunoglobulin superfamily
CSP: circumsporozoite protein
CSR: class switch recombination
CTSL: cathepsin L
DC: dendritic cell
EBV: Epstein-Barr virus
Ecb: extracellular-complement binding protein
Efb-C: extracellular fibrinogen-binding protein
ERK: extracellular signal related kinase
*F. tularensis*: *Francisella tularensis*
FB: factor B
FD: factor D
FH: factor H
FHA: filamentous hemagglutinin
FI: factor I
gC1qR: globular C1q receptor
g: gp, or GP, glycoprotein
GC: germinal center
HCV: hepatitis C virus
HHV-6: human herpes virus 6
HRgpA and RgpB: arginine-specific gingipain
HSV: herpes simplex virus
InlB: internalin B
IRF-1: interferon regulatory factor 1
*L. monocytogenes*: *Listeria monocytogenes*
LP: lectin pathway
*M. bovis* BCG: *Mycobacterium bovis Bacillus* Calmette-Guerin
*M. leprae*: *Mycobacterium leprae*
MAC: membrane attack complex
MAPK: mitogen-activate protein kinase
MASPs: MBL-associated serine proteases
MBL: mannose-binding lectin
NS: non-structural protein
*P. gingivalis*: *Porphyromonas gingivalis*
PGL-1: phenolic glycolipid 1
PI3K: phosphoinositide 3-kinase
PNH: paroxysmal nocturnal hemoglobinuria
RA: rheumatoid arthritis
*S. aureus*: *Staphylococcus aureus*
*S. pyogenes*: *Streptococcus pyogenes*
Sbi: *Staphylococcus* binder of IgG
SCIN: staphylococcal complement inhibitor
SLE: systemic lupus erythematosus
SpA: staphylococcal protein A
TCR: T cell receptor
Th: T helper cell
TLR: Toll-like receptor
Treg: regulatory T cell
USF-1: upstream stimulating factor 1
VCP: vaccinia control protein
WNV: West Nile virus
